# A new era in gammaherpesvirus transcriptomics: high-resolution profiling and model development

**DOI:** 10.1128/msystems.00208-25

**Published:** 2025-07-23

**Authors:** Kiran Fida, Brent A. Stanfield

**Affiliations:** 1 Department of Pathobiological Sciences, Louisiana State University School of Veterinary Medicine, Baton Rouge, Louisiana, USA; 2 Division of Biotechnology and Molecular Medicine (BioMMed), LSU-SVM, Baton Rouge, Louisiana, USA; University of Wisconsin-Madison, Madison, Wisconsin, USA

**Keywords:** guinea pig, gamma herpesvirus, small animal model, transcriptomics, CaGHV-1, Caviid gammaherpesvirus 1

## Abstract

A recent article by Torma et al. (G. Torma, Á. Dörmő, Á. Fülöp, D. Tombácz, et al., mSystems 10:e01678-24, 2025, https://doi.org/10.1128/msystems.01678-24) presents the first high-resolution, long-read transcriptomic atlas of Caviid gammaherpesvirus 1 (CaGHV-1), offering insights into its transcriptional complexity. Using nanopore direct RNA and cDNA sequencing, the study maps transcription start sites, polyadenylation signals, alternative splicing, and upstream open reading frames (uORFs), revealing a landscape of coding and non-coding RNAs with pervasive transcriptional overlaps. These features underscore the evolutionary conservation of key regulatory mechanisms across gammaherpesviruses, notably replication and transcription activator (RTA)-mediated transcriptional control. By establishing CaGHV-1 as a promising model for Kaposi’s sarcoma-associated herpesvirus (KSHV)-related disease, the work sets a foundation for studies of viral gene regulation, immune evasion, and pathogenesis, including bacterial artificial chromosomes (BACs) for genetic manipulation. Here, we discuss the advances in the study as well as how some limitations remain regarding read coverage biases, sequencing errors, and the uncharacterized functions of non-coding RNAs and emphasize the need for validation and functional assays. This study provides a valuable resource for understanding gammaherpesvirus biology and advancing translational research in viral pathogenesis and therapeutic development.

## COMMENTARY

A recent article by Torma et al. (1) marks a significant advancement in herpesvirus research by providing the first comprehensive, long-read sequencing-based transcriptomic atlas for Caviid gammaherpesvirus 1 (CaGHV-1). This research moves beyond basic transcript cataloging by achieving unprecedented resolution in mapping transcriptional start sites, polyadenylation signals, and alternative splicing events, which are critical for understanding the full complexity of CaGHV-1 gene regulation (2). These findings not only refine our understanding of gammaherpesvirus transcriptional architecture but also highlight CaGHV-1 as a powerful tool for modeling Kaposi’s sarcoma-associated herpesvirus (KSHV-related pathogenesis and developing targeted therapeutics (3).

Torma et al. used nanopore-based direct RNA sequencing (dRNA-Seq) and direct cDNA sequencing (dcDNA-Seq) to create a high-resolution atlas of the CaGHV-1 transcriptome. Nanopore sequencing is advantageous because it allows for the direct sequencing of native RNA molecules, thereby preserving post-transcriptional modifications and avoiding amplification biases inherent in other methods (4). This approach enabled the precise identification of transcriptional start and stop sites, alternative splice variants, and regulatory motifs. The study reveals that CaGHV-1 exhibits a remarkably rich and intricate transcriptional landscape, with extensive use of alternative splicing, transcript isoforms, and overlapping RNAs, which surpasses what has previously been described for non-human gammaherpesviruses.

The analysis not only identified the anticipated coding transcripts but also discovered numerous non-coding RNAs (ncRNAs), including polycistronic messages and replication origin-associated RNAs (raRNAs). These ncRNAs are crucial in the regulation of viral gene expression and host-virus interactions. Transcriptional overlaps—encompassing convergent, divergent, and co-oriented transcripts—are pervasive across the CaGHV-1 genome. This mirrors, yet also uniquely extends, the regulatory strategies reported in Kaposi’s sarcoma-associated herpesvirus (KSHV) and Epstein-Barr virus (EBV), indicating a conserved, yet distinct network of transcriptional regulation across gammaherpesviruses (2).

A pivotal discovery from the manuscript is the conservation of replication and transcription activator (RTA)–mediated transcriptional regulation: CaGHV-1’s ORF50, which encodes RTA, demonstrates key regulatory features akin to those in KSHV, suggesting evolutionary conservation not just in gene content but in molecular reactivation mechanisms (5). This finding is crucial because RTA is a master regulator of the lytic replication cycle in gammaherpesviruses, and its conservation suggests common mechanisms of viral reactivation. These findings set the stage for mechanistic studies of lytic reactivation and latency control using CaGHV-1 as an experimental system.

A notable aspect of this study is the identification of uORFs in CaGHV-1. While KSHV uORFs are positioned near ATG start codons, facilitating translational regulation (6). CaGHV-1 uORFs are located farther upstream. This suggests a distinct translational control strategy that may impact protein synthesis efficiency and viral gene expression. These findings offer an opportunity to explore how viral uORFs influence pathogenesis and immune evasion across different gammaherpesviruses.

## ESTABLISHING CAGHV-1 AS A PLATFORM FOR PATHOGENESIS MODELS

By documenting extensive parallels in transcriptional complexity and architecture with human disease-associated gammaherpesviruses, Torma et al. provide strong support for CaGHV-1 as a relevant model to dissect viral gene regulation, explore immune evasion tactics, and develop preclinical interventions for KSHV-related diseases. The RNA atlas serves as a crucial foundation for future functional and comparative virology studies and will likely facilitate the development of novel antiviral strategies.

## FUNCTIONAL EXPLORATION OF NON-CODING RNAs

While comprehensive sequencing confirms robust production of a diverse array of CaGHV-1 non-coding RNAs, their biological functions remain uncharacterized. Like KSHV, CaGHV-1 expresses numerous polycistronic and replication origin-associated RNAs, which are hypothesized to influence replication, immune evasion, and latency maintenance. However, the precise regulatory roles of these ncRNAs are yet to be discovered. The study of viral microRNAs and their functions has been extensively reviewed, highlighting their importance in modulating host-virus interactions and influencing viral pathogenesis (7). However, the precise regulatory roles of these ncRNAs are yet to be discovered.

Future research should center on elucidating the functions and mechanistic actions of these ncRNAs through discovery-driven approaches and functional assays (e.g., RNA immunoprecipitation, transcriptome-wide association studies). RNA immunoprecipitation followed by sequencing (RIP-Seq) is a powerful technique to identify RNAs that interact with specific RNA-binding proteins, thereby providing insights into the function of these ncRNAs. Determining whether CaGHV-1 ncRNAs interface with host immune response pathways—akin to the known activities of KSHV PAN RNA or viral miRNAs—will be essential for understanding viral persistence, reactivation, and potential oncogenicity.

## MODELING GAMMAHERPESVIRUS INFECTION AND DISEASE: FROM TRANSCRIPTOME TO PATHOGENESIS

The detailed transcriptomic map presented in the companion article provides a platform for translational studies leveraging CaGHV-1 in guinea pigs. Early *in vivo* work has shown that CaGHV-1 induces mild lymphoproliferative responses in its natural host, resembling mononucleosis-like syndromes, highlighting its biological relevance as a gammaherpesvirus (8). By coupling advanced transcriptomic profiling with targeted molecular tools (such as recombinant virus engineering), future studies can now dissect the pathogenesis, latency, and transformation potential of CaGHV-1 *in vivo*. Beyond recapitulating KSHV infection features, the development of a CaGHV-1 bacterial artificial chromosome (BAC), as outlined, will further empower studies of gene function, allow the creation of chimeric viruses, and accelerate translational research targeting KSHV-specific interventions—all made possible by the viral genomic and transcriptomic resources generated in the companion article. The construction of BACs will enable the manipulation of the viral genome, allowing for the creation of mutants and recombinant viruses to study gene function and pathogenesis (9).

## LIMITATIONS OF THE STUDY

Despite its contributions, the study has certain limitations that warrant consideration. While long-read sequencing provides superior transcript resolution, it is prone to sequencing errors and requires rigorous validation. The authors address this by integrating multiple sequencing approaches and bioinformatics pipelines; however, further validation through independent experimental methods could strengthen the conclusions. Additionally, the functional significance of many detected transcripts remains speculative. While the study suggests possible roles for these RNAs based on conservation and genomic context, further experimental validation is necessary to confirm their biological relevance. Moreover, detecting low-abundance transcripts via ONT remains difficult due to biases in read coverage. While direct RNA sequencing preserves RNA modifications, it has lower throughput and read quality compared with cDNA sequencing. Another challenge is read length variability, which complicates the distinction of isoforms and necessitates advanced bioinformatics tools for accurate interpretation (10).

## CONCLUSIONS

Torma et al. have set a new benchmark in gammaherpesvirus transcriptomics by mapping the full transcriptional architecture of CaGHV-1 using state-of-the-art long-read sequencing. Their findings redefine the scope of viral gene and ncRNA expression in animal models and establish CaGHV-1 as both a model of bio-complexity and a practical surrogate for dissecting KSHV biology. This work paves the way for future discovery and therapeutic validation in a mammalian host with greater translational relevance to human disease.
